# Clinical experience with radioactive iodine in the treatment of childhood and adolescent Graves' disease

**DOI:** 10.1530/EC-12-0049

**Published:** 2012-12-05

**Authors:** Adriano N Cury, Verônica T Meira, Osmar Monte, Marília Marone, Nilza M Scalissi, Cristiane Kochi, Luís E P Calliari, Carlos A Longui

**Affiliations:** 1 Pediatric Endocrinology Unit, Pediatrics Department Irmandade da Santa Casa de Misericórdia de São Paulo 01221-020, São Paulo Brazil; 2 Endocrinology and Metabolism, Medicine Department Irmandade da Santa Casa de Misericórdia de São Paulo 01221-020, São Paulo Brazil; 3 Nuclear Medicine Laboratory Irmandade da Santa Casa de Misericórdia de São Paulo 01221-020, São PauloBrazil

## Abstract

**Background/aims:**

Treatments for Graves' disease (GD) in children and adolescents include oral antithyroid drugs (ATDs), near total thyroidectomy, and radioactive iodine (RAI). ATDs remain the preferred choice in this age group, but because persistent remission occurs in 30% of cases, RAI is becoming a common option for definitive therapy.

**Methods:**

We performed a review of 65 medical records of GD patients under age 19 years who were followed between 1985 and 2005.

**Results:**

The prevalence of GD was higher in females (3:1) and during puberty (for both genders). If no remission was detected during ATD treatment, RAI was indicated when the following criteria were present: non-compliance, relapse, or side effects that were related to ATDs, large goiter, and long-term use of ATDs. The majority of patients developed hypothyroidism within 6 months after RAI. A progressive higher dose regimen was implemented in the last 10 years of the study period. A second RAI dose was necessary in eight cases. During the follow-up period, three pregnancies occurred. One patient with a thyroid nodule and benign cytology was detected.

**Conclusions:**

RAI therapy is effective and safe in the treatment of GD in children and adolescents.

## Introduction

Graves' disease (GD) accounts for 10–15% of all childhood thyroid abnormalities and is rare in those under age 5 years – its incidence peaks between age 11 and 15 years, predominantly affecting females (1, 2). There is tremendous controversy regarding the most appropriate treatment schedule for this age group (3). No strategy targets the underlying autoimmunity, and the control of thyroid hyperfunction is usually achieved by the induction of hypothyroidism.

In many countries, antithyroid drugs (ATDs) remain the first-line therapy (1, 2, 4, 5), but they have several drawbacks, such as a high prevalence of side effects (20–30%), prolonged need for oral therapy, and low remission and high relapse rates during or after discontinuation of ATDs (2, 6). Surgery is often indicated in Europe as a definitive therapy for GD, having the advantage of inducing rapid progression to hypothyroidism after total thyroidectomy, and is the second-line therapy after ATD. Conversely, it a surgical procedure that can effect complications, depending on the surgeon's experience (1). Radioactive iodine (RAI) therapy has been used frequently, especially in the USA (7), as an alternative first-line therapy to surgery and ATDs.

The incidence of adverse side effects of ATDs in young patients is high (3, 8) and is more frequent with propylthiouracil (PTU) (9, 10). Recently, an FDA report (PTU-induced hepatitis and acute liver failure) stated that PTU is not recommended as a first-line treatment in children (11). Thus, long-term experience with significantly more patients who undergo RAI therapy is needed to determine its safety profile in children and adolescents. There is also concern with regard to its potential carcinogenic effects and interference with reproductive function (3).

The purpose of this study was to evaluate the efficacy and long-term follow-up safety of RAI therapy in GD patients aged under 19 years. Like other centers, our protocol for ^131^I for GD is based on a fixed dose for all patients, aiming to induce remission of the disease and achieve hypothyroidism or euthyroidism (12).

## Patients and methods

We reviewed the medical records of 65 patients who were followed between 1985 and 2005 with clinical features and laboratory findings of hyperthyroidism due to GD. At the time that RAI was administered, the patients' ages ranged from 5 to 19 years.

The variables that we studied included associated diseases, gender, age, and stage of puberty (before, during, and after RAI treatment), ATDs that were given before RAI therapy, hormone concentrations, and imaging, such as ultrasonography and thyroid scan radioiodine uptake. Other data that were related to RAI therapy were total iodine dose, number of doses, and clinical progression after RAI, such as time to achieve hypothyroidism or euthyroidism.

## Results

**Figure 1:**
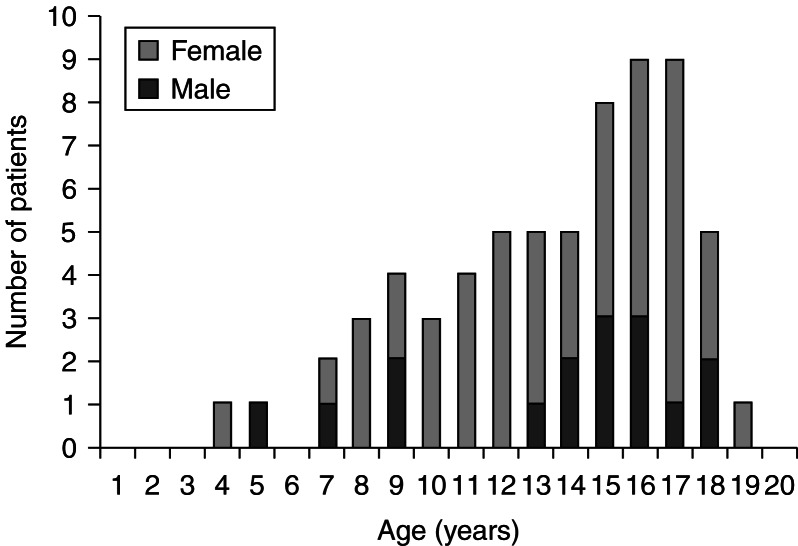
Age distribution of children at diagnosis of GD.

We studied 65 patients (49 females and 16 males; 3:1 ratio) with a mean (s.d.) age at diagnosis of 12.6 (3.6) years in females and 12.6 (4.8) years in males. At the time of admission to the pediatric endocrinology unit, the mean (s.d.) age was 13 (3.4) years in females and 14 (3.9) years in males (Fig. 1). Associated diseases were detected in five patients: Down syndrome (three cases), myasthenia gravis (one case), and nephrotic syndrome (one case).

The mean (s.d.) patient age when the first RAI dose was administered was 14.7 (3.1) years. Eight female patients needed a second RAI dose after 12 months because they remained in a state of hyperthyroidism, as confirmed by biochemical analysis. It took longer than the usual 6-month interval for the second dose to be given due to a lack of availability of service (tertiary hospital with work overload).

At the time that the RAI dose was administered, 31/49 (64.5%) females were at Tanner stage V of puberty in the breast, 11/49 (23.0%) were between stages bII and bIV, and 6/49 (12.5%) were at the prepubertal stage. Among male patients, 9/16 (60.0%) were at genital stage gV, 3/16 (20.0%) were between stages gII and gIV, and 3/16 (20.0%) were prepubertal. During the follow-up after RAI, 11 females and 2 males completed pubertal development.

Oral ATDs were the initial treatment in 61/65 patients who received RAI. Four patients discontinued ATD treatment after medical prescription, not for any adverse side effect, and at the time for RAI was not under ATD use. PTU was the sole drug in 27/61 patients vs methimazole (MTZ) in 19/61, and alternate use of PTU and MTZ was implemented in 15/61 patients for various treatment periods. The choice of drugs varied, based on availability at the primary care facility at which patients retrieved their medications. Treatment period of ATD therapy was 16.9 (11.4) months. Levothyroxine (l-T_4_) was used in all patients who progressed to hypothyroidism after RAI therapy. No patient was subjected to ‘block and replace’ treatment.

The chief criteria that were used to prescribe RAI were presence of large goiter (16 cases (24.6%)), inadequate clinical control of GD during ATD treatment (27 cases (41.5%)), irregular use of ATD (ten patients (15.4%)), hyperthyroidism relapse after ATD discontinuation (six cases (9.3%)), presence of ATD-related adverse side effects (three cases (4.6%) – two with hepatitis and one with leukopenia), and other indications, such as financial limitations and long periods of ATD treatment (three cases (4.6%)).

**Figure 2:**
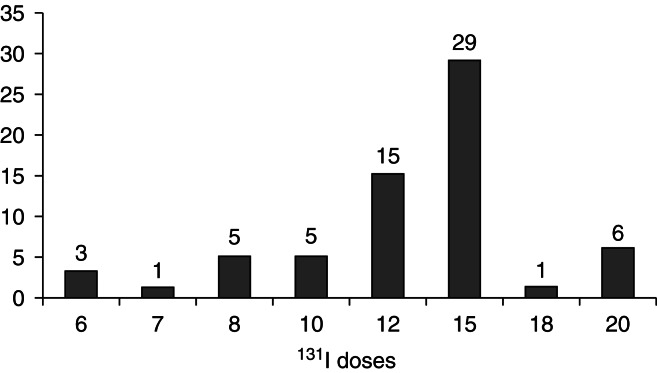
Distribution of RAI doses in 65 patients with GD.

**Figure 3:**
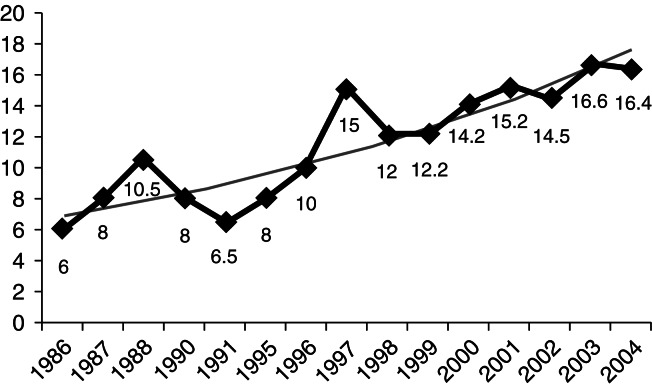
Average RAI dose during the study period.

The initial dose of RAI was 6 mCi; 14 patients (21.5%) were given doses between 6 and 10 mCi, 44 (67.7%) were treated with doses that ranged from 12 to 15 mCi, and 7 (10.8%) were given doses that exceeded 15 mCi but were below 20 mCi (Fig. 2). Eight female patients needed a second RAI dose, which varied between 15 and 25 mCi. During the entire observation period, we observed a progressive increase in RAI dose (Fig. 3), using a fixed dose for all patients and considering RAI uptake and gland size. When we refer to ‘fixed dose’, we are describing the prescribed dose, which can be changed by the nuclear medical service, based on thyroid uptake and gland size, explaining the variation in dose in certain cases.

**Figure 4:**
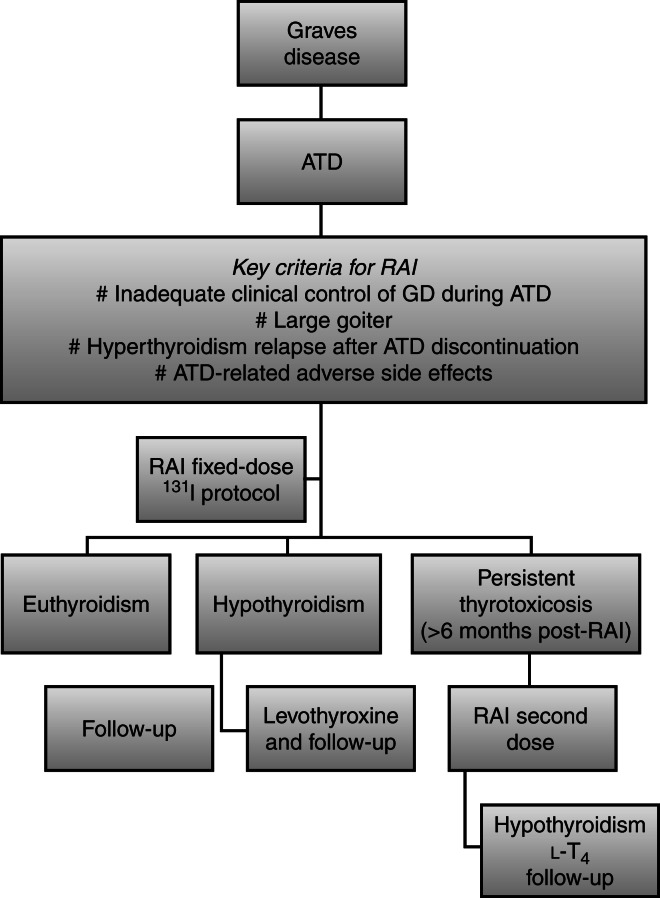
Treatment and outcomes in pediatric population with Graves' disease. ATDs, antithyroid drugs; l-T_4_, levothyroxine.

On evaluation of clinical progression after RAI therapy, we noted 52/65 (80.0%) patients who developed hypothyroidism, of whom 16 (30.0%) did so within 3 months after RAI, 25 (48.0%) within 6 months, 3 (5.7%) within 6 and 12 months, and 8 (15.3%) patients >12 months. Eight of 65 (12.3%) patients had euthyroidism 6 months after RAI therapy and 5/65 (7.9%) patients did not achieve remission 7 months after RAI therapy. A diagnosis of hypothyroidism was based on TSH and free T_4_ levels at 3, 6, and 12 months after RAI. Patients with euthyroidism were followed every 6 months and no thyroid cancer has been diagnosed during follow-up in all patients (Fig. 4).

Thyroid ultrasonography was available for 29 (44.6%) patients before RAI to identify increased thyroid volume or absence of nodules. The 24-h thyroid radioiodine uptake was measured in 24 patients, revealing a mean (s.d.) of 55.4% (19.6); thyroid scans were unavailable for all patients in their medical records and were not required for a diagnosis of GD. Thyroid function was available for all 65 patients and antithyroid antibodies for 45 patients before RAI (Table 1).

The duration of follow-up after RAI ranged from 7 to 24 years (mean (s.d.), 11.5 (3.6)), and no late effects of ^131^I, such as thyroid carcinoma and gonadal and hematological abnormalities, were observed. One patient was diagnosed with a thyroid nodule during a long follow-up, but a cytological analysis confirmed a benign subtype (adenoma).

## Discussion

RAI therapy has been used for several years in the treatment of GD in not only adulthood but also childhood and adolescence. Many studies in this age group have reported adequate efficacy and safety (13, 14, 15). Treatment of children with RAI can affect remission rates that exceed 95% (3, 16, 17).

Using a fixed-dose ^131^I protocol, when a dosimetry reading of thyroid volume is unavailable, a relative dose of 220–275 μCi/g thyroid tissue (200–250 Gy) usually leads to hypothyroidism (18), and except for larger goiters, which need a higher dose of ^131^I, a total dose of 12–15 mCi will promote remission (19). Over the past 20 years, most of our subjects received ^131^I doses of 12–15 mCi (Fig. 2) and developed hypothyroidism.

The duration of follow-up after RAI was similar to that in other studies that failed to observe any increased risk in thyroid malignancy (3, 16) in certain subjects who were followed for more than 20 years in the pediatric clinic without any evidence of thyroid carcinoma, as some groups have reported (16).

Consistent with previous data, most of our patients were female adolescents (1, 20, 21). Considering the safety recommendations regarding the use of RAI for GD in children and adolescents (1, 3, 22), our youngest patient was 5 years old (23), although RAI therapy is usually prescribed at the end of pubertal development. In our series, progression to pubertal Tanner stage V was adequate after RAI, and the secretion of gonadotropins was normal. The few pregnancies (3/49 females) and abortions (1/3 pregnancies) did not allow us to draw a conclusion on the long-term safety with regard to fertility. Nevertheless, RAI therapy has no significant adverse effects on fertility rates or on the offspring of children and adolescents, such as congenital anomalies, who have been treated with RAI (16, 24).

Antithyroid medications are the typical first-line treatment for GD in children and adolescents (1, 3, 25, 26); in our study, ATD was the preferred treatment in most patients. Pretreatment with ATD did not appear to alter the efficacy or outcome after RAI therapy, and there was no relationship between ATD use and an increased need for a second dose of radioiodine, consistent with earlier reports (16, 27, 28, 29).

In this study, our criteria for prescribing RAI therapy were those that are usually used (1, 3, 6, 17, 30, 31), including a lack of clinical control, irregular use of ATD, relapse after ATD discontinuation, presence of side effects of ATDs, absence of remission during long-term treatment with ATDs, and presence of a large goiter – <80 g when surgery is the initial treatment (32). Notably, our objective was not to correlate prior use of ATDs with outcome of RAI therapy.

Nearly all patients progressed to hypothyroidism at rates of 75–90%. In a recently published meta-analysis of 1874 patients in 29 trials, the overall cure rate of RAI reached 49.7% (37.8% of hypothyroidism cases) as first-, second-, or third-line therapy (5). Hypothyroidism was usually detected in the first year, especially in the first 6 months post-RAI.

The efficacy of radioiodine is dose dependent, but in contrast to other reports (33), none of the patients who needed a second dose received an inadequately low first dose of RAI. As suggested by Kraiem & Newfield (1), the requirement for a second RAI dose is related to individual differences in thyroid sensitivity to radiation. Despite the dietary recommendations before RAI, we cannot rule out the possibility of thyroid contamination with nonradioactive iodine, which can lead to minor thyroid radioiodine uptake. We noted an increase in RAI dose during observation, likely reflecting the rise in dietary iodine in the general population (33). One limitation was the absence of urinary excretion of iodine, which was unable to be measured.

There is concern regarding the greater risk of relapse in non-Caucasian patients, which who are ∼2.5 times more likely to suffer a relapse than Caucasian patients (2, 34). Our results underscore the value of RAI therapy in children and adolescents with GD, which should be considered a first-line therapy, taking into account ethnicity (our entire study group was non-Caucasian), increases in T_4_ and TRAb serum levels (10-unit increase) (34), and lower age (35). Another limitation was that in several cases, TRAb was not verified; TRAb has been fully available in our workup only in the last 5–10 years. As reported by McIver & Morris (36), we observed associated diseases, such as Down syndrome, nephrotic syndrome, and myasthenia gravis, requiring the pediatrician to be aware of these concomitant diseases.

We acknowledge several limitations our study. It was a retrospective study that included patients over 22 years (1986–2008), explaining the absence of autoantibody analysis in some subjects. Moreover, we do not routinely assess ioduria, due to lack of laboratory availability. We also chose a fixed-dose protocol because we were unable to perform dosimetry for all patients.

In conclusion, treatment with radioiodine is highly effective in the short term and can be used safely during childhood and adolescence.

## Author contribution statement

A N Cury, V T Meira, O Monte, M Marone, N M Scalissi, C Kochi, L E P Calliari, and C A Longui contributed equally to this work.

## Figures and Tables

**Table 1:** Clinical and biochemical profile at diagnosis GD in pediatric population.

	**Mean (s.d.) or percentage**	***n***
Age (years)	12.6 (3.8)	65
Female/male	75/25	65
Prepubertal	14/86	65
TSH (0.5–5.3 μU/ml)	0.2 (0.4)	62
Tri-iodothyronine (94–241 ng/dl)	442.1 (241.8)	57
T_4_ (6.4–13.3 μg/dl)	25.7 (10.7)	56
FT_4_ (0.7–1.6 ng/dl)	6.1 (2.8)	54
TPOAb positive (>50 IU/ml)	87%	45
TgAb positive (>50 IU/ml)	56%	43
